# IgM kappa proliferative glomerulonephritis with monoclonal immunoglobulin deposition complicated with nocardiosis dermatitis: a case report and review of literature

**DOI:** 10.3389/fmed.2024.1161560

**Published:** 2024-04-12

**Authors:** Kebao Chen, Yue Wang, Jinyu Yu, Xueyao Wang, Zhonggao Xu, Yanbo Li, Weixia Sun

**Affiliations:** Department of Nephrology, The First Hospital of Jilin University, Changchun, China

**Keywords:** proliferative glomerulonephritis, monoclonal immunoglobulin deposition, IgM kappa, nocardiosis dermatitis, MGRS

## Abstract

**Rationale:**

Monoclonal gammopathy of renal significance (MGRS) represents a group of disorders caused by monoclonal immunoglobulin (M protein) secreted by B cells or plasma cells. Proliferative glomerulonephritis with monoclonal immunoglobulin deposition (PGNMID) is a glomerular disease and a form of MGRS. Here, we presented a rare case of a patient with IgM kappa PGNMID complicated with nocardiosis dermatitis.

**Patient concerns and diagnoses:**

A 56-year-old man was admitted to the hospital because of cutaneous purpura and proteinuria. His initial pathological diagnosis indicated membranous proliferative glomerulonephritis, IgM(++), and subacute interstitial nephritis. Based on further examination, he was finally diagnosed to have IgM kappa PGNMID and subacute interstitial nephritis. After the initial diagnosis, the patient received hormonal therapy. During the treatment, nocardiosis dermatitis emerged as a complication, and the hormonal therapy was gradually reduced. The patient refused further treatment with rituximab, and his health is currently stable.

**Outcomes:**

IgM kappa PGNMID complicated with nocardiosis dermatitis is an extremely rare occurrence. Laboratory examination and pathological analysis are required to confirm the diagnosis of this disorder. Timely and accurate diagnosis is essential for the appropriate treatment of PGNMID.

## Introduction

Proliferative glomerulonephritis with monoclonal immunoglobulin deposition (PGNMID) is a rare variant of monoclonal gammopathy of renal significance (MGRS), and its pathogenesis is caused by abnormal monoclonal immunoglobulin (mIg) deposition in the glomerulus (1, 2). MGRS is characterized by pathogenic monoclonal immunoglobulins or light chains produced by pre-malignant plasma cells or B cell clones. Amyloidosis is the main type of MGRS, followed by MIDD, cryoglobulinemia, and PGNMID. The most common isoform involved in PGNMID is IgG (mainly IgG3), and in rare cases, it is IgA or IgM isoform. This disease was first reported in 2004 (2, 3). The main manifestations of the disease are proteinuria, hematuria and renal insufficiency in different degrees, and more than 50% of patients with PGNMID eventually progress to chronic renal failure (4, 5). The diagnosis is based on the examination of clinical manifestations, renal pathology, immunology, and hematology. Besides, other mIg-related glomerular diseases need to be excluded, such as type I cryoglobulinemia, amyloidosis, etc. At present, the best treatment for this disease is not clear, and it is often advocated to rely on targeted and identifiable B cells and plasma cell clone-oriented therapy in clinic (2, 6). Here, we described a rare case of IgM kappa-type PGNMID complicated with nocardiosis dermatitis.

Most serum IgM belongs to the class of natural IgM, which is produced by B1 B cells and B1-derived plasma cells independent of antigen-encounter (7, 8). IgM polymers have 10 (pentamer) or 12 (hexamer) antigen-binding domains and consist of pairs of heavy chains with four constant domains, each with a variable domain paired with the corresponding light chain (9, 10). Unlike hexamer IgM antibodies, pentamers also include a j-chain that stabilizes the pentamer structure and binds to the corresponding receptor, such as the polymerized immunoglobulin receptor (pIgR) (11, 12). When IgM is deposited in the kidney, it can activate the local complement pathway by binding with specific epitopes of damaged or stressed glomerulus and C1q, causing the release of inflammatory factors (such as IL-6 and TNF-a), destroying the glomerular filtration barrier and leading to proteinuria. Here, we describe a rare case of IgM kappa PGNMID complicated with nocardia dermatitis. At present, the consensus of PGNMID treatment needs a clone-oriented method. However, some cases are stable for a long time after being treated with traditional methods. We report here a case of IgM kappa PGNMID whose urine protein and serum creatinine were stable for a long time after steroid and symptomatic treatment, and nocardia dermatophytosis was complicated in the course of the disease due to various factors.

## Case presentation

In November 2020, a 56-year-old man was hospitalized at the First Hospital of Jilin University because of cutaneous purpura and proteinuria that had occurred 1 year ago. The patient was suffering from purpura in both lower limbs without any apparent inducing factor. He visited the Second Hospital of Changchun City, where his proteinuria status and 24 h urinary protein level were found to be “+” and 0.4 g/day, respectively. The patient also showed gross hematuria and intermittent remission or new onset of purpura due to irregular treatment. During the follow-up, his 24 h urinary protein level increased to 2.63 g/day. One month ago, he had orally taken 3 capsules of Bailing Capsule, 3 pouches of Shenyanling Granules, and 2 pouches of Huaiqihuang Granules. During the disease course, he had cough and expectoration; however, he did not have fever, nausea, vomiting, joint swelling and pain, oral and nasal ulcers, hair loss, photosensitivity, dry mouth, and dry eyes. He also denied a history of hypertension, diabetes, and coronary heart disease. He had undergone appendectomy for treating appendicitis 40 years ago. He was a current smoker, with a history of smoking for more than 30 years at an average of 10 cigarettes per day.

Physical examination on admission revealed the following findings: temperature, 36.5°C; respiratory rate, 18 breaths/min; blood pressure, 160/91 mmHg; pulse rate, 90 bites/min; jugular vein, not distended; breathing sound in both lungs, pronounced; wet rales, audible. Moreover, he had mild edema and no abnormality in the heart and abdomen and no rash on both lower limbs. Laboratory investigation upon the patient’s first admission was shown in Table 1.

**Table 1:** Initial laboratory investigation upon admission.

Parameters	Value	References	Unit
24 h urinary protein	1.29	0–0.2	g
24 h urinary microalbumin	1448.4	0–30	mg
Serum creatinine	94.6	57–97	μmol/L
eGFR	77.17	>90	mL/min
Total protein	55.5	65–85	g/L
Serum albumin	33.4	40–55	g/L
White blood cell count	5.02	3.50–9.50	×10^9^/L
Red blood cell count	3.05	4.30–5.80	×10^12^/L
Hemoglobin	94	130–175	g/L
Platelet	209	125–350	×10^9^/L
Cholesterol	3.85	2.6–6.0	mmol/L
Triglyceride	2.19	0.28–1.80	mmol/L
Acid	262	210–430	μmol/L
Erythrocyte sedimentation rate	41	0–15	mm/h
Total IgG	5.61	8.6–17.4	g/L
C3	0.58	0.7–1.4	g/L
C4	0.05	0.1–0.4	g/L
IgM	4.81	0.3–2.2	g/L
IgA	1.04	1.0–4.2	g/L

Because the patient reported intermittent skin purpura in both lower limbs, he underwent the following examinations: three rheumatism tests; five immunological assays for IgG, IgA, IgM, and complement C3 and C4; rheumatoid factor (RF) typing; anticyclic citrullinated peptide (CCP) antibody test; antinuclear antibody (ANA) series test; antineutrophil cytoplasmic antibody (ANCA) test; and screening tests for autoimmune diseases. Serum immunoelectrophoresis assay showed positive results for IgM kappa light chain, while urine immunoelectrophoresis showed negative results. Because the patient showed positive result for serological tests related to rheumatism, increased RF level, decreased complement C3 and C4 levels, and a history of skin purpura, a cryoglobulin test was performed; however, the result was negative. A urological examination with an abdominal color Doppler ultrasonography revealed stones in the left kidney and prostate and hyperplasia of prostate. In line with the cough and expectoration symptoms and long-term smoking history of the patient, lung computed tomography (CT) showed slight chronic inflammation in the middle lobe of the right lung, the lingual lobe of the left lung, and a small nodular density shadow was seen in the middle lobe of the right lung, slightly pulling the adjacent pleura. The chronic inflammation in both lungs at the time of admission was due to high absolute value of neutrophils, symptoms of cough and expectoration, and long-term smoking history. The presence of lesions in the middle lobe of the right lung was considered to be due to previous untreated tuberculosis. The purified protein derivative test and the T-SPOT. TB test were performed to detect tuberculosis; however, both tests showed negative results.

**Figure 1:**
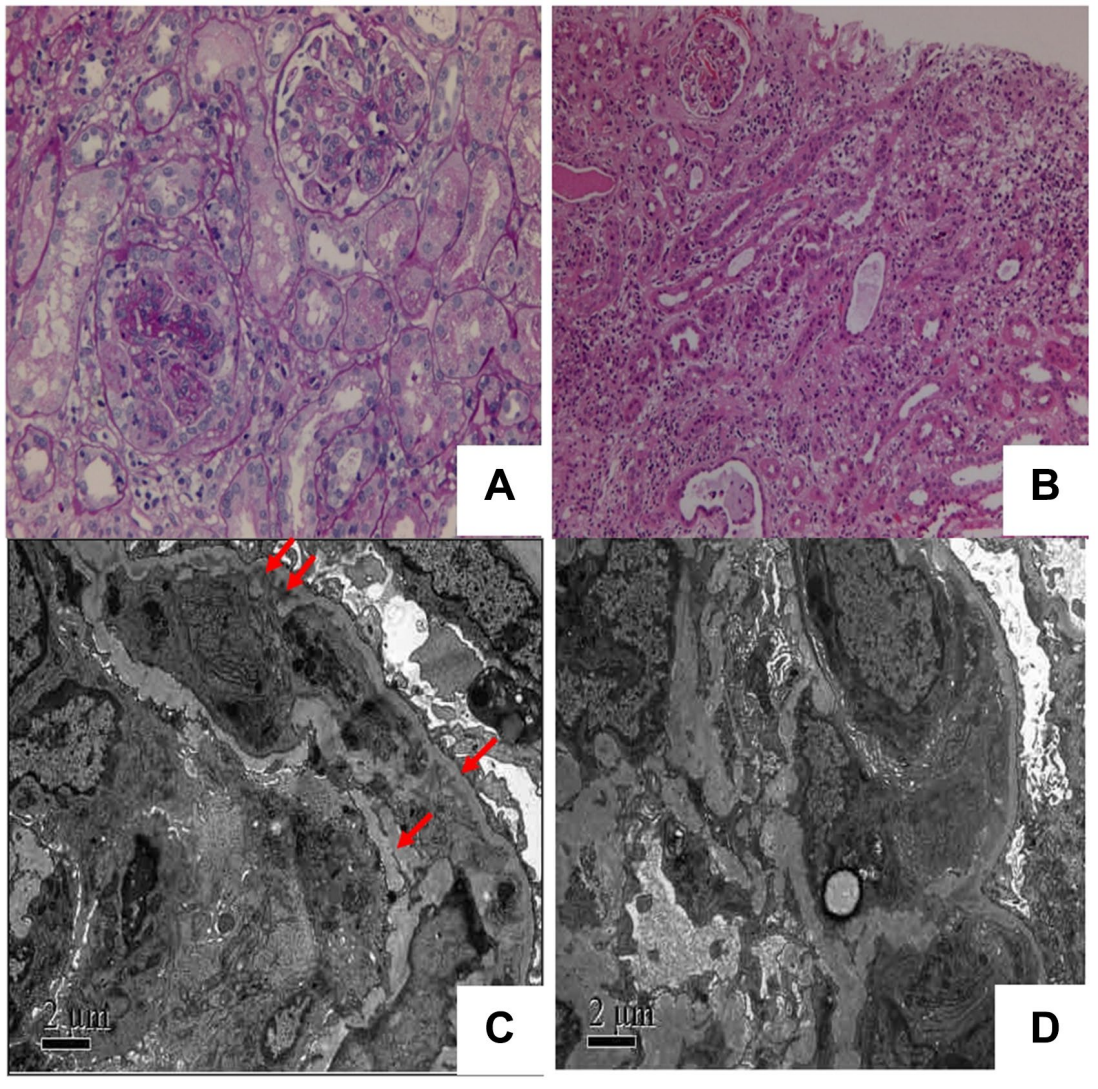
**(A)** Light microscopy shows segmental thickening of the glomerular basement membrane with segmental double-track sign (Periodic acid-Schiff staining: magnification ×100). **(B)** Light microscopy shows infiltraton of interstitial inflammatory cells (hematoxylin-eosin staining: 200× magnification ×100). **(C)** Electron microscopy shows the deposition of a massive electron-dense substance and the formation of a double-track sign in the subcutaneous layers (red arrow). **(D)** Electron microscopy shows that the cells in segmental capillaries were increased and swollen with lysosomes.

Kidney biopsy performed on December 1, light microscopy revealed that 2 of 21 glomeruli in the punctured renal tissue had glomerulosclerosis. In the remaining glomeruli mesangial cells diffuse proliferation with mesangial matrix segmental expansion, mesangial interposition into the capillary walls, and segmental swelling of the endothelial cells was found (Figure 1A). The localized focal renal interstitium showed lymphoid and macrophage infiltration and mild fibrosis (Figure 1B). The arterioles showed no apparent abnormalities. Congo red staining of the renal puncture tissue was negative. Immunofluorescence assay revealed segmental deposition of IgA(−), IgM(++), IgG(−), C3(++ to +++), C4(−), C1q(−), F(−), Kappa(−) and Lambda(−) along the glomerular capillary wall of five glomeruli. Membranoproliferative glomerulonephritis (MPGN) with mild subacute interstitial nephritis was the most probable diagnosis. Electron microscopy of the glomeruli revealed segmental insertion, segmental endothelial cell hyperplasia and swelling with an increased amount of lysosomes, bulk electron-dense deposition in the segmental subendothelium and subsegmental epithelium, no specific tangible structures, and segmental thickening of the glomerulus basement membrane with segmental double track sign (Figure 1C). Podocyte foot processes were fused. Renal tubular epithelial cells showed vacuolar degeneration and an increase in the amount of lysosomes; moreover, some of these cells were atrophied. Interstitial edema, infiltration of lymphocytes and mononuclear cells, and collagen fiber hyperplasia were observed (Figure 1D). Except for subsequent nephritis, the diagnosis was consistent with MPGN with tubulointerstitial damage.

Serum IgM kappa immunoelectrophoresis was positive and serum protein electrophoresis showed the presence of M protein in serum. Therefore, hematological diseases were not excluded, and bone needle biopsy was performed. The bone marrow test indicated the possibility of secondary anemia and showed that 1% percent of the plasma cells in the bone marrow were naïve. It should be noted that multiple myeloma cannot be identified through hematological tests, and patients are advised to undergo periodical reexaminations.

The patient showed repeated occurrence of purpura in both lower limbs during the disease. However, because there was no occurrence of purpura during hospitalization and no photos of skin purpura were provided, a skin biopsy was not performed, and the nature of purpura, such as allergic purpura or Henoch-Schönlein purpura dermatitis, could not be determined. Based on the results of renal biopsy and related examinations, the patient was diagnosed to have MPGN, subacute interstitial nephritis, hypertension, and moderate anemia. Furthermore, according to the lung CT findings and tuberculosis-related indicators, after consultation with a hospital specializing in tuberculosis treatment, it was considered that the patient’s tuberculosis condition was stable and noninfectious; therefore, anti-tuberculosis treatment was presently not required. As the patient had subacute renal tubulointerstitial injury, glucocorticoid therapy was recommended after a complete assessment of the patient’s condition, and the patient and his family were informed of the side effects of this therapy. After obtaining consent, prednisone acetate (40 mg) was administered orally once daily from December 5, 2020. At discharge, the patient’s serum creatinine level was 144.2 μmol/L, and the 24 h urinary protein level was 1.14 g/day. He returned to our hospital for a re-examination on January 6, 2021. The immunological assay results were as follows: decreased levels of total IgG and complement C4 (5.21 g/L and 0.02 g/L, respectively), normal levels of IgA and complement C3 (1.03 g/L and 0.7 g/L, respectively), and increased levels of IgM (6.52 g/L). Serum immunoelectrophoresis assay revealed positive results for IgM and kappa light chain. The laboratory tests revealed the following results: increased 24 h urinary protein level (2.44 g/day), decreased eGFR (74.31 mL/min), increased serum creatinine level (97.6 μmol/L), and decreased hemoglobin level (108 g/L). After treatment, the serum creatinine level was stable, and anemia was corrected, thus demonstrating the effectiveness of hormonal therapy. The dose of prednisone acetate was reduced to 35 mg/day orally, and then glucocorticoid administration gradually decreased, and it was reduced to 25 mg orally once daily on March 8, 2021.

**Figure 2:**
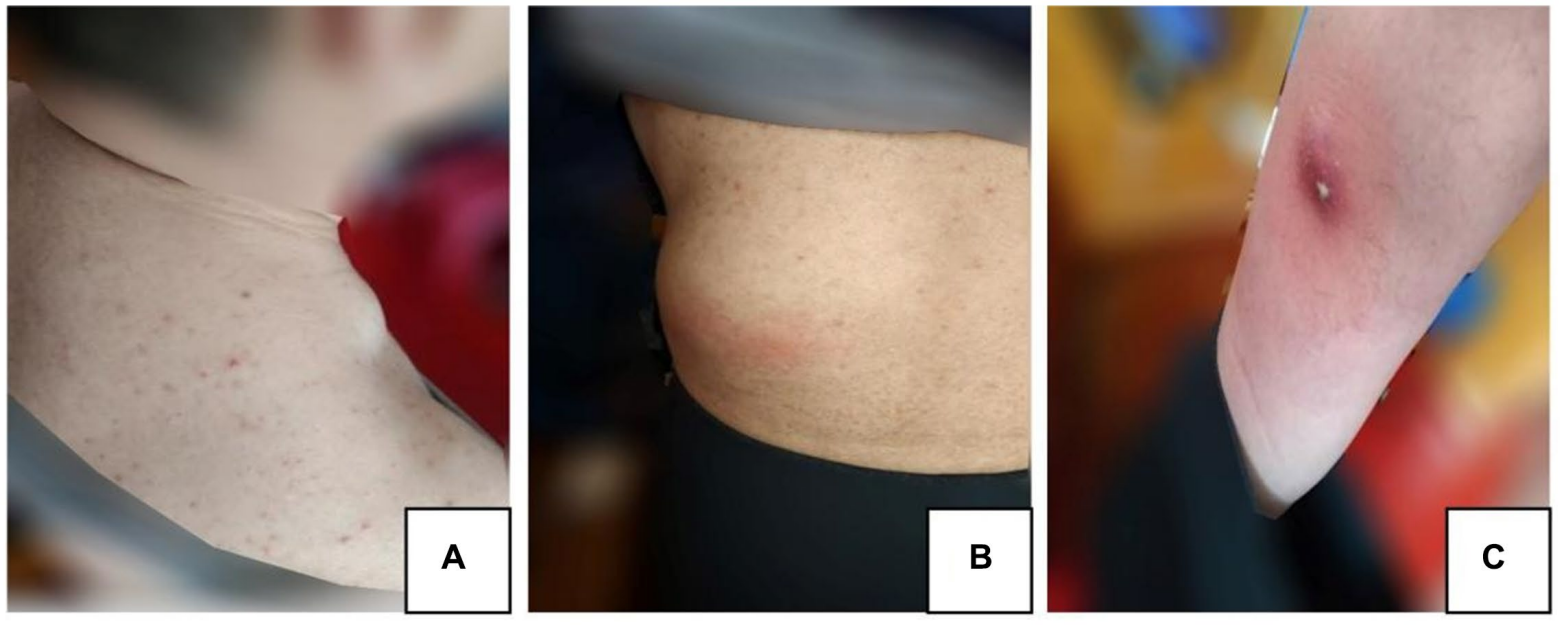
**(A)** Neck mass. **(B)** Lumbar mass. **(C)** Anterior tibia mass.

On March 20, 2021, the patient began to experience pain and showed development of skin masses on the neck, lumbar region, and anterior tibia (Figures 2A–C). A lymph node color Doppler ultrasound and tuberculin test revealed no abnormalities. A tissue biopsy revealed a necrotic mass, and the tumor showed no improvement even after oral administration of moxifloxacin hydrochloride tablets (Baifule). In mid-April, he traveled to Shanghai for medical treatment, where a bone marrow biopsy was performed to rule out hematological malignancies. An ultrasonography of the tumor in our hospital in late April revealed that the cyst was mixed with a solid tumor, and the largest tumor in the waist measured was 44 mm × 61 mm. The lung CT scan showed a small amount of tuberculosis in the middle lobe of the right lung; however, this finding was uncertain. A closer examination of the density shadow of the soft tissue at the root and axilla of the left neck was recommended. The T-SPOT. TB test showed a negative result, and a consultation with the tuberculosis-specializing hospital confirmed that the patient had stable and noninfectious tuberculosis. On April 27, 2021, the daily dose of prednisone acetate was lowered to 20 mg orally. On April 30, 2021, the patient experienced discomfort and fever, indicating changes in the characteristcs of the tumor. Cutaneous abscess puncture drainage was performed in the Otolaryngology Department of The Second Hospital of Jilin University. Second-generation sequencing of the abscess drainage fluid detected 58,017 sequences of *Nocardia* and 6 sequences of human cytomegalovirus (CMV). Experts from the Department of Infectious Diseases confirmed the diagnosis of cutaneous nocardiosis and did not rule out human CMV infection. From May 7, 2021, the patient was given 2 tablets of compound sulfamethoxazole orally twice a day; ganciclovir (75 mg) and ampicillin sodium (4 g) were administered intravenously twice a day; and the dose of prednisone acetate was lowered to 10 mg/day. He was admitted to the Second Hospital of Jilin University on May 10, 2021. The findings on admission were as follows: blood pressure, 122/82 mm Hg; creatinine level, 201 μmol/L; β2-microglobulin level, 5.34 mg/L; albumin level, 29.8 g/L; white blood cell count, 19.4 × 10^9^/L; and neutrophil count, 18.7 × 10^9^/L. Prednisone acetate, Betrolek, Bailing capsules, and Huaiqihuang granules were prescribed as medications for the MPGN. Anti-infection treatment was commenced with compound sulfamethoxazole and minocycline hydrochloride pills, while antiviral treatment was provided through intravenous administration of ganciclovir and ampicillin. The patient’s hormone levels gradually decreased. Albumin supplementation was initiated on May 18, 2021, at the frequency of one bottle/day. On May 24, 2021, the patient’s symptoms considerably improved; his creatinine level decreased to 150 μmol/L and total white blood cell count reduced to 6.1 × 10^9^/L. The tumor drainage strip was removed on May 28, 2021. On May 31, 2021, he was discharged from the hospital under the following conditions: creatinine, 145 μmol/L; β2-microglobulin, 4.0 mg/L; eGFR, 45.7 mL/min; and normal total white blood cell and neutrophil counts. Treatment with sulfamethoxazole and amoxicillin-clavulanate potassium orally was continued for 3 months following discharge.

**Figure 3:**
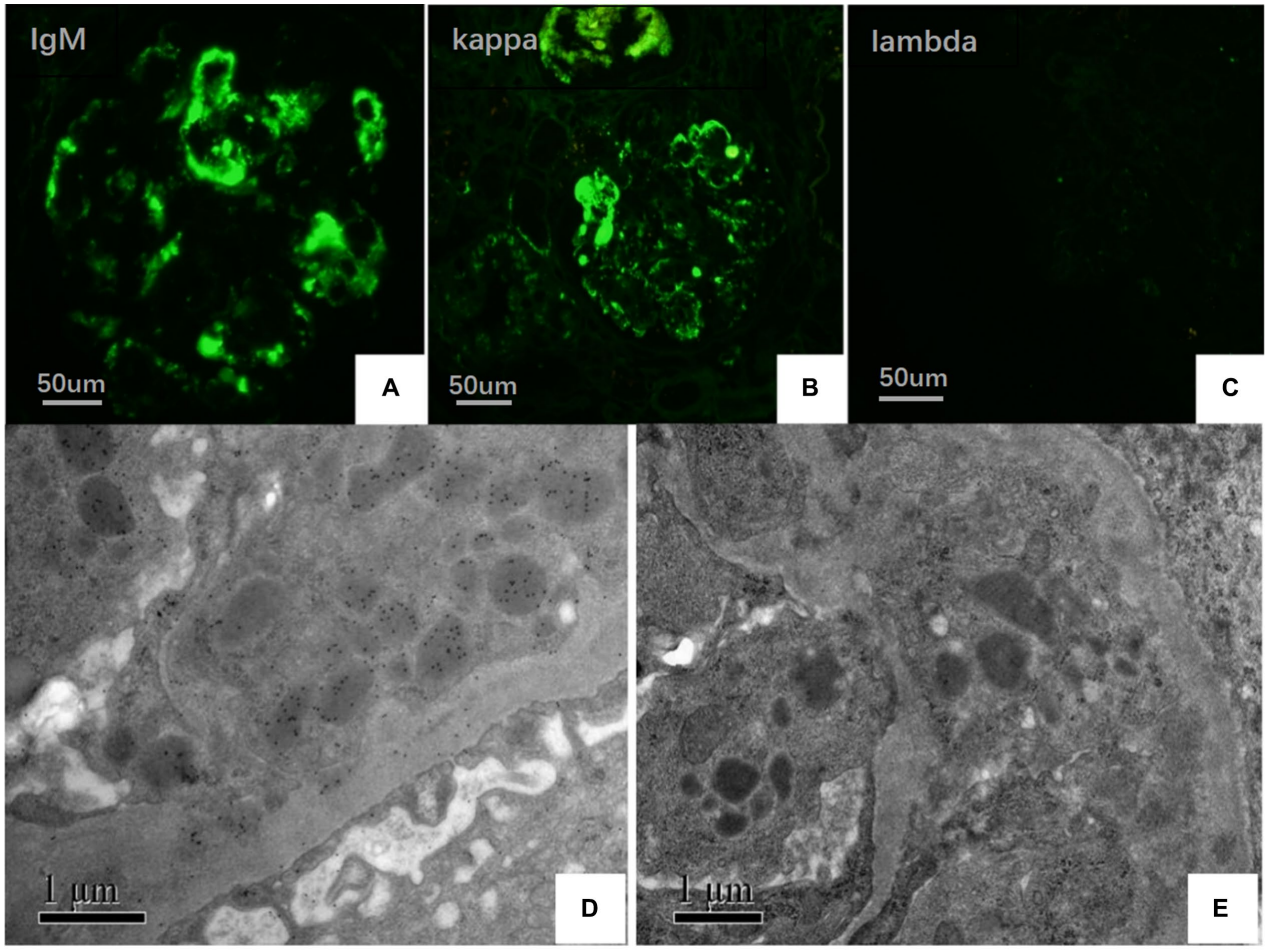
**(A–C)** Under IF, IgM and kappa light chain were deposited in mesangial area and vascular loops (green), where lambda light chain was negative (FITC direct immunofluorescence:magnification ×400). **(D)** Electron microscopy showed kappa light chain (++ to +++) in glomerular subendothelial and subepithelial deposition. **(E)** Electron microscopy shows lambda light chain (±).

**Figure 4:**
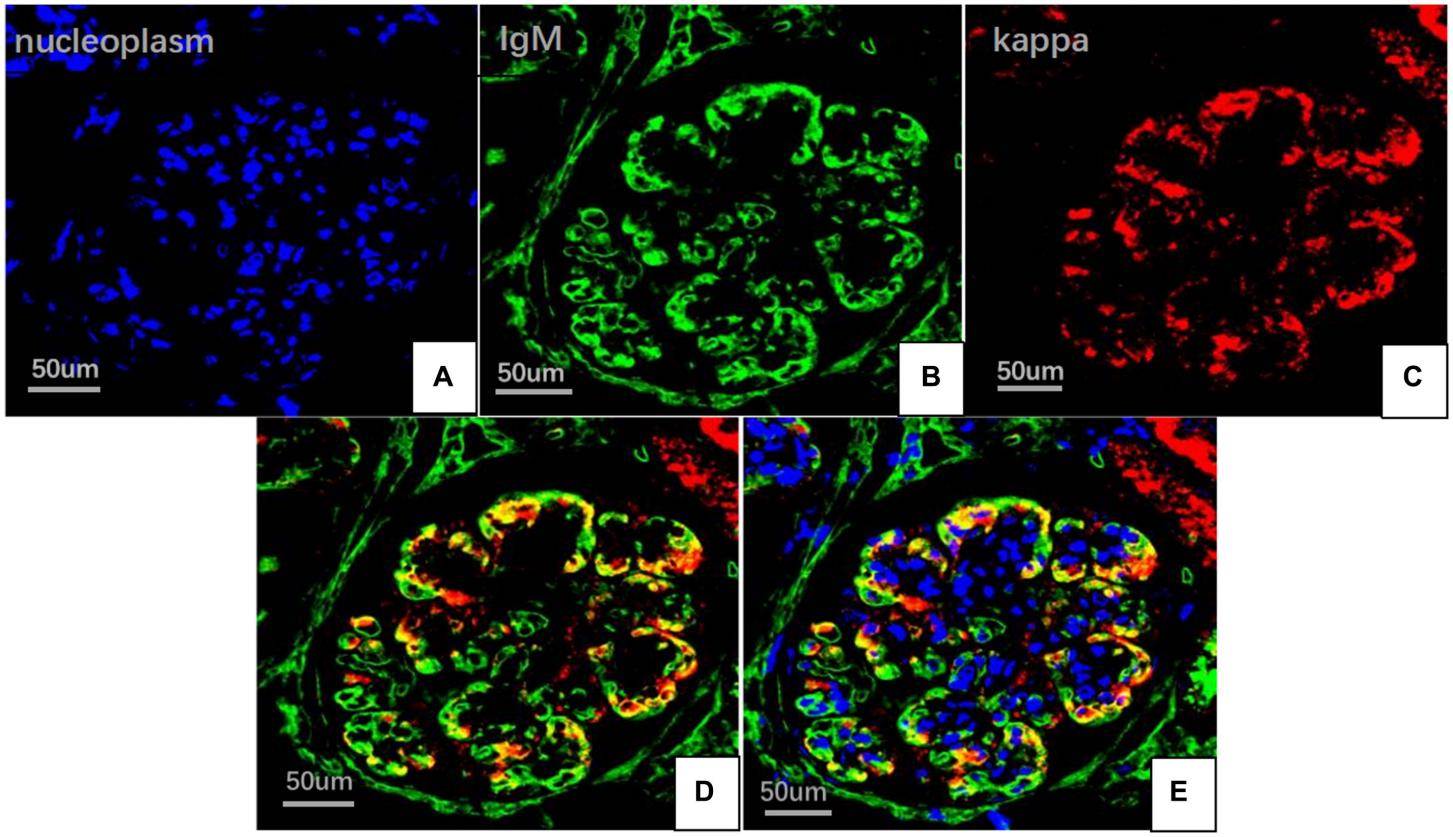
Multiplex immune fuorescence staining of glomerular (FITC indirect immunofluorescence:magnification ×400). **(A)** Nucleoplasm (blue). **(B)** IgM light chainstaining (green). **(C)** Kappa light chain (red). **(D)** IgM and kappa light chain. **(E)** IgM, kappa light chain and nucleoplasm.

**Figure 5:**
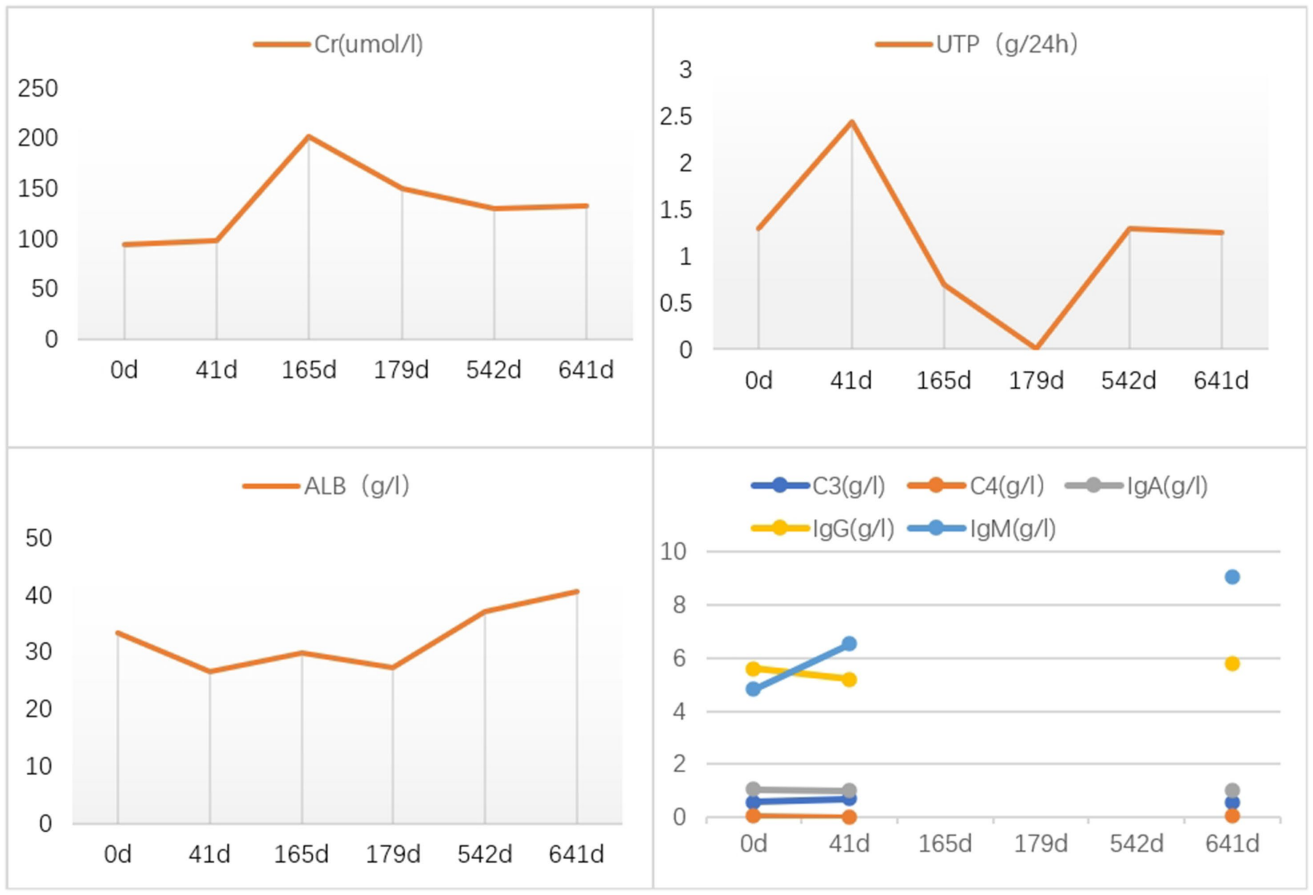
Clinical course of the patient after admission. Creatinine (Cr); 24 h urinary protein quantification (UTP); serum albumin (ALB); complement 3 (C3); complement 4 (C4); immunoglobin A (IgA); total immunoglobin G (IgG); immunoglobin M (IgM).

On May 22, 2022, the following findings were noted at our hospital: serum creatinine level, 130.3 μmol/L; 24 h urinary protein level, 1.29 g/day; eGFR, 51.65 mL/min; and albumin level, 37.2 g/L. The neutrophil and total white blood cell counts were also normal. Serum Ig light chain and IgM levels were elevated. Paraffin-embedded renal tissue sections were used for antigen repair and immunofluorescence assay. The following results were obtained: IgA (−), IgM (++), IgG (−), C3 (++ to +++), C4 (−), C1q (−), F (−), kappa (++), and lambda (±) (Figures 3A–C). Immunoelectron microscopy suggested that glomerular subendothelial and subepithelial deposition and presence of lysosomes in endothelial cells, with kappa (++ to +++) and lambda (±) (Figures 3D,E). This finding confirmed the diagnosis of PGNMID with restricted expression of kappa light chains. On August 29, 2022, the patient’s visit at our hospital yielded the following results: serum creatinine level, 133 μmol/L; 24 h urinary protein level, 1.242 g/day; and albumin level, 40.7 g/L. Serum immunoelectrophoresis yielded positive results for IgM and kappa light chain. The results for the five immunity factors were as follows: total IgG, 5.81 g/L; IgA, 1 g/L; IgM, 9.06 g/L; complement C3, 0.55 g/L; and complement C4, 0.06 g/L. At the same time, we performed multiple immunofluorescence staining, and in this case, IgM and kappa light chain co-located well (Figures 4A–E). The patient was finally diagnosed to have IgM kappa-type PGNMID. Figure 5 shows the clinical course of the patient. Rituximab was recommended as the next therapeutic option; however, the patient and his family declined to opt for this therapy.

## Discussion

MGRS is a disorder caused by monoclonal immunoglobulins secreted by clones of nonmalignant plasma cells or B cells (13). PGNMID is a specific type of MGRS (14). Kidney biopsies often show mesangial proliferative, membranoproliferative, or intracapillary proliferative glomerulonephritis on light microscopy, and the deposits are usually monoclonal Ig; this disorder was first reported in 2004 (3). Usually, the deposition of IgG, IgA, and IgM is a rare incidence (2, 3, 15–19). In the present case, kappa light chain deposition was initially not detected in the glomerulus by fluorescence staining of the frozen sections, and the diagnosis was not confirmed. After immunofluorescence assay and immunoelectron microscopy of paraffin sections were performed, the kappa chain deposition was confirmed. Further multiple immunofluorescence staining showed that IgM and kappa light chain were well co-located, indicating that monoclonal IgM was deposited in kidney tissue. Taken together with the results of serological tests and light microscopy, the patient was diagnosed to have IgM kappa PGNMID. Immunofluorescence on frozen sections (IF-F) is the traditional pathological test for diagnosing kidney diseases; however, the use of frozen sections of a kidney biopsy has limitations and can affect the diagnosis of the disease when frozen tissue is inadequate or unavailable (20, 21). Moreover, according to previous studies, the determination of Ig deposition with the IF-F method may yield false negative result, which is termed “masked deposits,” and the reason for this issue is related to the destruction of antigens in frozen sections (21–23). Paraffin immunofluorescence (IF-P) is a valuable salvage technique, as antigen retrieval in IF-P staining can unmask immune deposits; thus, IF-P is more sensitive than IF-F in some renal diseases such as PGNMID (24). Recent studies show that RF IgM has different affinities. Similar to natural autoantibodies found in healthy people, the low-affinity RF IgM is polyreactive and reduces the level of disease-causing IgG autoantibodies in the body by neutralizing IgG (25). However, RF IgM with high affinity is monoreative, which can protect the antigen it recognizes from degradation. RF IgM autoantibodies from rheumatoid arthritis patients typically bind with high affinity the Fc portion of IgG, which may protect and hence intensify the effect of pathogenic IgG (25, 26). Therefore, it is of great significance to identify the polyreactive and monoreactive of IgM deposited in the kidney tissue of this patient to understand the pathogenic role of RF IgM in this patient. Due to the lack of renal tissue samples in this case, IgM reactivity could not be identified.

IgM kappa light chain-type PGNMID is extremely rare. To date, only 7 cases have been reported in PubMed and other databases; of these, 6 cases were due to unknown causes, and 1 case was caused by hematological malignancies (see Table 2). The majority of patients with PGNMID are middle-aged and older males, and therapeutic options include glucocorticoids, bortezomib, rituximab, and novel cloning-directed therapy. Based on previous studies, the pathogenesis of PGNMID may be associated with hematological malignancies, infections, and immunocompromised patients (2, 27–30). In the present case, testing such as bone marrow aspirate biopsy ruled out hematological malignancies. Fujita et al. (31) reported two cases of PGNMID in patients infected with parvovirus B19. The clinical symptoms of PGNMID subsided after antiviral therapy, and laboratory testing revealed normal condition, thus suggesting that PGNMID in these two individuals was caused by the viral infection (2, 31). The causative factor of this rare disorder is unknown, and the pathogenic factors of this rare case are still unclear, and no hematological malignant tumor has been found in clinic. After 4 months of glucocorticoid therapy, the patient showed infection with *Nocardia farcinica*, and laboratory tests showed no substantial improvement in the patient’s condition even after this infection was treated. This finding indicates that infection is not the direct cause of PGNMID, but rather a coexisting condition that occurs during its therapy.

**Table 2:** Summary of previous reports of IgM Kappa PGNMID and their clinical characteristics.

References	Years	Age	Gender	Treatment plan	Curative effect
van Kruijsdijk (5)	2020	53/34	Female/Female	Azathioprine + prednisolone/bortezomib + rituximab + dexamethasone (later discontinued due to side effects)	Serum creatinine remained stable, no hematuria or proteinuria/serum creatinine remained stable, no hematuria or proteinuria
Yamaguchi (8)	2018	53	Male	Angiotensin receptor blocker	After 3 years, eGFR decreased slightly (65.7 to 64.2) and urinary protein increased (3.5 to 3.8 g/g)
Yahatam (10)	2012	38	Male	Steroids + prednisone + angiotensin receptor blockers + statins	Proteinuria and serum creatinine levels remained unchanged, and serum creatinine levels gradually increased to 1.5 mg/dL after prednisone was discontinued
Oe (11)	2013	48	Male	(Secondary to chronic lymphocytic leukemia) rituximab + fludarabine + cyclophosphamide	Protein/creatinine ratio decreased by 2 g/g, serum creatinine did not change significantly
Lima (12)	1990	76	Male	6-methylprednisolone + chlorosuccinic acid	Serum creatinine remained stable and 24 h urine protein was less than 1 g
Ramosr (13)	2007	48	Male	Prednisone + chlorbutanol	After 1 year of treatment, monoclonal m and k chains disappeared in serum and urine immunofixation, serum IgM levels remained high, proteinuria remained unchanged, and renal function deteriorated

In the present case, the patient showed many skin abscesses during steroids therapy. Together with Nocardia sequences, the sequences of human CMV were also detected in the abscess drainage fluid. Human CMV infection is a severe complication in immunocompromised patients. Skin manifestations of this infection are very rare; maculopapules are the main manifestation on the skin, and ulcers, blisters, erosion, plaques, and nodules may also appear (32, 33). The probability of skin abscesses is very low, and most of the lesions are located in the perianal, genital, and the surrounding areas (34). This patient showed no skin rash, ulcer, blisters, or other manifestations, and the abscesses were distributed in the neck, lumbar region, and anterior tibia. Because the number of sequences of *Nocardia* in the skin abscess drainage fluid was significantly higher than that of human CMV, *Nocardia* infection was consider the cause of abscess. Nocardiosis is a rare disease caused by *Nocardia*, which occurs as respiratory or skin infections; most patients have lung infections, followed by central nervous system and skin infections (35). It often occurs in immunodeficient patients (HIV infection, etc.) or immunocompromised patients (long-term use of glucocorticoids, malignant tumors, diabetes, etc.). The lung CT findings of the patient were normal, and there was no manifestation of central nervous system infection; thus, the diagnosis was confirmed as nocardiosis dermatitis. The cause of infection with this bacterial species is considered to be related to the patient’s history of nephritis, treatment with glucocorticoids, and low immunity. Sulfonamides are the primary first-line treatment agents for nocardiosis, and trimethoprim-sulfamethoxazole (TMP-SMX) is the most widely used sulfonamide (36). The patient’s condition improved after receiving treatment for nocardiosis, including puncture, drainage, and compound sulfamethoxazole. When the first medication is ineffective, combination therapy—such as amikacin and TMP-SMX, imipenem and TMP-SMX, or amikacin and cefotaxime—is frequently used (36, 37). Interferon, a new class of medication that is currently being used to treat refractory disseminated *Nocardia* infection, functions primarily by inducing several antibacterial effects and by inhibiting the intracellular growth of pathogens. Although it has a good therapeutic effect, it is rarely used in clinical practice because of several negative side effects (38).

PGNMID was previously treated mainly through empirical treatment, including steroids and immunosuppressive drugs (such as cyclophosphamide), and some patients achieved temporary relief. In recent years, bortezomib and rituximab have been used for treating PGNMID (39). The former can stabilize renal function by inducing apoptosis of monoclonal plasma cells and inhibiting renal fibrosis. The latter is an anti-CD20 monoclonal antibody that protects the kidneys by depleting B lymphocytes and thereby improves the disease remission rate (19, 39, 40). In recurrent cases, adequate immunosuppression, such as immunosuppressants combined with rituximab, should be selected (17, 19, 40, 41). The latest approach to treat PGNMID is clone targeting, represented by daratumumab, which is commonly used in treating multiple myeloma and mainly targets abnormal plasma cell clones. However, in only 30% of patients with PGNMID, abnormal monoclonal immunoglobulins can be detected in serum, urine, or bone marrow; consequently, daratumumab has not been widely used (18, 42).

Based on the diagnosis and treatment of this case and the review of relevant literature, we found that the incidence of PGNMID was low and that IgM kappa-type PGNMID with the complication of nocardiosis dermatitis was even rarer. This rare condition must be differentiated from glomerular disorders with deposits of monoclonal Igs (e.g., amyloidosis, type I cryoglobulinemia-induced renal damage, etc.). In the present case, bone and lymph node biopsies ruled out hematologic illnesses; however, the underlying etiology is unknown. Currently, the patient’s condition is stable, and he declined to undergo rituximab treatment. Our case suggested that physicians should be vigilant of this illness, perform prompt diagnosis, and offer the patient the best course of therapy.

## Data availability statement

The original contributions presented in the study are included in the article/supplementary material, further inquiries can be directed to the corresponding authors.

## Ethics statement

Written informed consent was obtained from the individual(s) for the publication of any potentially identifiable images or data included in this article.

## Author contributions

KC, YW, and XW were involved in patient management and data collection. JY performed the pathological analysis of the patient. ZX conducted statistical analysis of the data. KC and WS contributed to study design, data analyses, and writing of the manusctipt. All authors contributed to the article and approved the submitted version.

## References

[1] Sethi S Rajkumar SV D'agati VD. The complexity and heterogeneity of monoclonal immunoglobulin-associated renal diseases. J Am Soc Nephrol. (2018) 29:1810–23. doi: 10.1681/ASN.2017121319, PMID: 29703839 PMC6050917

[2] Li M Xu G. An update of proliferative glomerulonephritis with monoclonal immunoglobulin deposits. Clin Kidney J. (2022) 15:1041–8. doi: 10.1093/ckj/sfab269, PMID: 35664272 PMC9155251

[3] Nasr SH Markowitz GS Stokes MB Seshan SV Valderrama E Appel GB. Proliferative glomerulonephritis with monoclonal IgG deposits: a distinct entity mimicking immune-complex glomerulonephritis. Kidney Int. (2004) 65:85–96. doi: 10.1111/j.1523-1755.2004.00365.x, PMID: 14675039

[4] Ogura Y Yabushita S Aihara H Tsukada H Hashiba T Furuse S. A case of proliferative glomerulonephritis with monoclonal IgG deposits (PGNMID) that responded favorably to steroid therapy. CEN Case Rep. (2022) 11:208–15. doi: 10.1007/s13730-021-00653-3, PMID: 34628583 PMC9061924

[5] Lin L Chen N. A review on the diagnosis and treatment of proliferative glomerulonephritis with monoclonal immunoglobulin deposits. Int J Gen Med. (2022) 15:8577–82. doi: 10.2147/IJGM.S386733, PMID: 36540764 PMC9760043

[6] Zhou H Li M Zeng C Chen Z Zhang T Cheng Z. Efficacy of immunomodulatory drugs in combination with dexamethasone in proliferative glomerulonephritis with monoclonal immunoglobulin deposits. Kidney Int Rep. (2022) 7:2166–75. doi: 10.1016/j.ekir.2022.07.009, PMID: 36217516 PMC9546741

[7] Boes M. Role of natural and immune IgM antibodies in immune responses. Mol Immunol. (2000) 37:1141–9. doi: 10.1016/S0161-5890(01)00025-6 11451419

[8] Baumgarth N. The double life of a B-1 cell: self-reactivity selects for protective effector functions. Nat Rev Immunol. (2011) 11:34–46. doi: 10.1038/nri2901, PMID: 21151033

[9] Pan S Manabe N Yamaguchi Y. 3D structures of IgA, IgM, and components. Int J Mol Sci. (2021) 22:12776. doi: 10.3390/ijms222312776, PMID: 34884580 PMC8657937

[10] Eskeland T TB C. IgM molecules with and without J chain in serum and after purification, studied by ultracentrifugation, electrophoresis, and electron microscopyn. Scand J Immunol. (1975) 4:217–28. doi: 10.1111/j.1365-3083.1975.tb02620.x 807966

[11] Li Y Wang G Li N Wang Y Zhu Q Chu H. Structural insights into immunoglobulin M. Science. (2020) 367:1014–7. doi: 10.1126/science.aaz5425, PMID: 32029689

[12] Kubagawa H Oka S Kubagawa Y Torii I Takayama E DW K. The long elusive IgM Fc receptor, FcμR. J Clin Immunol. (2014) 34:S35–45. doi: 10.1007/s10875-014-0022-7 24793544 PMC4160156

[13] GnanasampanthanSKousiosA. Monoclonal gammopathies of renal significance. Clin Med. (2023) 23:250–3. doi: 10.7861/clinmed.2023-RM337236803

[14] Leung N Bridoux F Batuman V Chaidos A Cockwell P D’Agati VD. The evaluation of monoclonal gammopathy of renal significance: a consensus report of the International Kidney and Monoclonal Gammopathy Research Group. Nat Rev Nephrol. (2018) 15:45–59. doi: 10.1038/s41581-018-0077-4 PMC7136169 30510265

[15] Leung N Drosou ME Nasr SH. Dysproteinemias and glomerular disease. Clin J Am Soc Nephrol. (2018) 13:128–39. doi: 10.2215/CJN.00560117, PMID: 29114004 PMC5753301

[16] Kanzaki G Okabayashi Y Nagahama K Ohashi R Tsuboi N Yokoo T. Monoclonal immunoglobulin deposition disease and related diseases. J Nippon Med Sch. (2019) 86:2–9. doi: 10.1272/jnms.JNMS.2019_86-1 30918151

[17] van Kruijsdijk RCM Abrahams AC Nguyen TQ Minnema MC JFM J Limper M. Clone-directed therapy for proliferative glomerulonephritis with monoclonal immunoglobulin depositions: is it always necessary?: Two case reports and literature review. J Nephrol. (2020) 33:611–7. doi: 10.1007/s40620-020-00723-2, PMID: 32221859 PMC7220881

[18] Hogan JJ Alexander MP Leung N. Dysproteinemia and the kidney: core curriculum 2019. Am J Kidney Dis. (2019) 74:822–36. doi: 10.1053/j.ajkd.2019.04.029, PMID: 31331759

[19] Xu ZG Li WL Wang X Zhang SY Zhang YW Wei X. Proliferative glomerulonephritis with monoclonal immunoglobulin G deposits in a young woman: a case report. World J Clin Cases. (2021) 9:2357–66. doi: 10.12998/wjcc.v9.i10.2357, PMID: 33869614 PMC8026847

[20] Larsen CP Ambuzs JM Bonsib SM Boils CL Nicholas Cossey L Messias NC. Membranous-like glomerulopathy with masked IgG kappa deposits. Kidney Int. (2014) 86:154–61. doi: 10.1038/ki.2013.548, PMID: 24429395

[21] Larsen CP Boils CL Cossey LN Sharma SG Walker PD. Clinicopathologic features of membranous-like glomerulopathy with masked IgG kappa deposits. Kidney Int Rep. (2016) 1:299–305. doi: 10.1016/j.ekir.2016.08.012, PMID: 29142932 PMC5678740

[22] Larsen CP Messias NC Walker PD Fidler ME Cornell LD Hernandez LH. Membranoproliferative glomerulonephritis with masked monotypic immunoglobulin deposits. Kidney Int. (2015) 88:867–73. doi: 10.1038/ki.2015.195, PMID: 26154922 PMC4687465

[23] Nasr SH Fidler ME Said SM. Paraffin immunofluorescence: a valuable ancillary technique in renal pathology. Kidney Int Rep. (2018) 3:1260–6. doi: 10.1016/j.ekir.2018.07.008, PMID: 30450452 PMC6224795

[24] Santoriello D Nasr SH. Novel approaches beyond standard immunofluorescence for kidney biopsies. Curr Opin Nephrol Hypertens. (2022) 31:221–7. doi: 10.1097/MNH.0000000000000783, PMID: 35256574

[25] Nicolò A Amendt T El Ayoubi O Young M Finzel S Senel M. Rheumatoid factor IgM autoantibodies control IgG homeostasis. Front Immunol. (2022) 13:1123117. doi: 10.3389/fimmu.2022.1016263 36341420 PMC9634112

[26] Yaku A Ishikawa Y Iwasaki T Hiwa R Matsuo K Saji H. Genetic architecture underlying IgG-RF production is distinct from that of IgM-RF. Rheumatology. (2023) 62:2015–20. doi: 10.1093/rheumatology/keac593 36250908

[27] Yamaguchi Y Maeda K Nagatoya K Yamauchi A. A case report of proliferative glomerulonephritis with monoclonal immunoglobulin M-kappa deposits without associated lymphoproliferative disorder or detectable paraproteinemia. CEN Case Rep. (2018) 7:55–61. doi: 10.1007/s13730-017-0291-0, PMID: 29230710 PMC5886923

[28] Aucouturier P D'agati VD Ronco P. A fresh perspective on monoclonal gammopathies of renal significance. Kidney Int Rep. (2021) 6:2059–65. doi: 10.1016/j.ekir.2021.04.026, PMID: 34386655 PMC8343799

[29] Liu M Yu X Wang S Qin A Zhou F Zhao M. Proliferative glomerulonephritis with monoclonal immunoglobulin deposits: an entity associated with distinct diseases and comparison between IgG1 and IgG3 subtypes. J Nephrol. (2022) 35:2363–72. doi: 10.1007/s40620-022-01317-w, PMID: 35460458

[30] Oshio M Fujii T Kusaura T Nagahama K. Relapsing proliferative glomerulonephritis with monoclonal IgG deposits showing circumferential crescentic glomerulonephritis. Clin Kidney J. (2013) 6:635–8. doi: 10.1093/ckj/sft121, PMID: 26069833 PMC4438369

[31] Fujita E Shimizu A Kaneko T Masuda Y Ishihara C Mii A. Proliferative glomerulonephritis with monoclonal immunoglobulin G3kappa deposits in association with parvovirus B19 infection. Hum Pathol. (2012) 43:2326–33. doi: 10.1016/j.humpath.2012.04.004, PMID: 22819999

[32] Neumann ABF Daxbacher ELR Chiaratti FC Jeunon T. Cutaneous involvement by cytomegalovirus in a renal transplant recipient as an indicator of severe systemic infection. An Bras Dermatol. (2016) 91:80–3. doi: 10.1590/abd1806-4841.20163989, PMID: 26982783 PMC4782651

[33] Choi YL Kim JA Jang KT Kim DS Kim WS Lee JH. Characteristics of cutaneous cytomegalovirus infection in non-acquired immune deficiency syndrome, immunocompromised patients. Br J Dermatol. (2006) 155:977–82. doi: 10.1111/j.1365-2133.2006.07456.x, PMID: 17034528

[34] Struble EB Murata H Komatsu T Scott D. Immune prophylaxis and therapy for human cytomegalovirus infection. Int J Mol Sci. (2021) 22:8728. doi: 10.3390/ijms22168728, PMID: 34445434 PMC8395925

[35] Mcphee L Stogsdill P Vella JP. *Nocardia farcinica* pericarditis after kidney transplantation despite prophylaxis. Transpl Infect Dis. (2009) 11:448–53. doi: 10.1111/j.1399-3062.2009.00413.x, PMID: 19508699

[36] Wilson JW. Nocardiosis: updates and clinical overview. Mayo Clin Proc. (2012) 87:403–7. doi: 10.1016/j.mayocp.2011.11.016, PMID: 22469352 PMC3498414

[37] Derungs T Leo F Loddenkemper C Schneider T. Treatment of disseminated nocardiosis: a host-pathogen approach with adjuvant interferon gamma. Lancet Infect Dis. (2021) 21:e334–40. doi: 10.1016/S1473-3099(20)30920-8, PMID: 34425068

[38] Bridoux F Javaugue V Nasr SH Leung N. Proliferative glomerulonephritis with monoclonal immunoglobulin deposits: a nephrologist perspective. Nephrol Dial Transplant. (2021) 36:208–15. doi: 10.1093/ndt/gfz176, PMID: 33494099

[39] Oki R Unagami K Taneda S Takagi T Ishida H. Treatment with bortezomib for recurrent proliferative glomerulonephritis with monoclonal IgG deposits in kidney allograft. Case report and review of the literature. J Nephrol. (2022) 35:1289–93. doi: 10.1007/s40620-022-01332-x, PMID: 35522429 PMC9107408

[40] Maan D Clark B Bunker M Arora S. Successful management of proliferative glomerulonephritis with monoclonal immune deposits with combined immunosuppressive therapy. BMJ Case Rep. (2018) 11:e225205. doi: 10.1136/bcr-2018-225205 PMC6301545 30567197

[41] Sawada A Kawanishi K Horita S Koike J Honda K Ochi A. Proliferative glomerulonephritis with monoclonal immunoglobulin G deposits complicated by immunoglobulin A nephropathy in the renal allograft. Nephrology. (2016) 21:48–52. doi: 10.1111/nep.12775 26971743

[42] Zand L Rajkumar SV Leung N Sethi S El Ters M Fervenza FC. Safety and efficacy of daratumumab in patients with proliferative GN with monoclonal immunoglobulin deposits. J Am Soc Nephrol. (2021) 32:1163–73. doi: 10.1681/ASN.2020101541, PMID: 33685975 PMC8259683

